# Correlation between Mitochondrial Dysfunction, Cardiovascular Diseases, and Traditional Chinese Medicine

**DOI:** 10.1155/2020/2902136

**Published:** 2020-10-07

**Authors:** Li Zhu, Zhigang Chen, Keli Han, Yilin Zhao, Yan Li, Dongxu Li, Xiulong Wang, Xuefang Li, Siyu Sun, Fei Lin, Guoan Zhao

**Affiliations:** ^1^The First Affiliated Hospital of Xinxiang Medical University, Xinxiang 453100, Henan, China; ^2^Henan Joint International Research Laboratory of Cardiovascular Injury and Repair, Xinxiang, Henan, China; ^3^Henan Engineering Research Center for Mitochondrion Biomedical of Heart, Xinxiang, Henan, China

## Abstract

Cardiovascular disease (CVD) is the number one threat that seriously endangers human health. However, the mechanism of their occurrence is not completely clear. Increasing studies showed that mitochondrial dysfunction is closely related to CVD. Possible causes of mitochondrial dysfunction include oxidative stress, Ca^2+^ disorder, mitochondrial DNA mutations, and reduction of mitochondrial biosynthesis, all of which are closely related to the development of CVD. At present, traditional Chinese medicine (TCM) is widely used in the treatment of CVD. TCM has the therapeutic characteristics of multitargets and multipathways. Studies have shown that TCM can treat CVD by protecting mitochondrial function. Via systematic literature review, the results show that the specific mechanisms include antioxidant stress, regulation of calcium homeostasis, antiapoptosis, and regulation of mitochondrial biosynthesis. This article describes the relationship between mitochondrial dysfunction and CVD, summarizes the TCM commonly used for the treatment of CVD in recent years, and focuses on the regulatory effect of TCM on mitochondrial function.

## 1. Introduction

With the continuous progress in the treatment of infectious diseases and the extension of human life span, the battlefield between humans and diseases has shifted to chronic noncommunicable diseases. Among them, cardiovascular disease (CVD) has become the leading cause of death in China and worldwide as its incidence continues to increase, and it poses a serious threat to the safety and quality of life of patients [1, 2]. Atherosclerosis, hypertension, myocardial ischemia-reperfusion injury, and heart failure are common CVDs or pathological processes. However, the mechanism of their occurrence is not completely clear. An increasing number of studies has shown that mitochondrial dysfunction is closely related to CVD. The mechanisms mainly include oxidative stress disorder, calcium disorder, reduction of mitochondrial biosynthesis, transition of mitochondrial permeability, and accumulation of mitochondrial DNA mutation. At present, TCM, which has the characteristics of multitargets and multipathways, is widely used in the treatment of CVD [3]. Therefore, in this paper, we discuss the relationship between mitochondrial dysfunction and CVD, as well as the therapeutic mechanism of TCM in the treatment of CVD with respect to mitochondrial function.

## 2. Functional Properties of Mitochondria

Mitochondria are semiautonomous organelles with a unique genetic system that provide the chemical energy required for biosynthesis, respiration, secretion, and mechanical movement in organisms; they are also important organelles that generate intracellular free radicals and regulate apoptosis [4–6]. Mitochondria are known as “capacity factories,” “apoptosis switches,” and “enzyme bags.” They are also called “cellular energy-processing factories” because they oxidize three major nutrients to provide adenosine triphosphate (ATP), which is required for life activities [4]. In addition to being energy producers, mitochondria are also the main site of reactive oxygen species [6] (ROS) production. Furthermore, mitochondria also play an important role in the regulation of intracellular calcium homeostasis, calcium-sensitive enzyme activity, and signal transduction [7]. In conclusion, mitochondria are central mediators of energy production, signal transduction, oxidative stress, Ca^2+^ homeostasis, and apoptosis regulation. Therefore, the normal function of mitochondria is of great importance in life activities.

## 3. Mitochondria and Cardiomyocytes

Cardiomyocytes are highly dependent on aerobic oxidation to supply energy. They contain a considerable amount of mitochondria, up to 20–30% of cell capacity, which provide more than 90% of energy to the heart muscle [8, 9]. The sources of myocardial energy include fatty acids, glucose, and other carbohydrates. These substrates are metabolized in mitochondria, providing energy for cardiomyocytes through oxidative phosphorylation. In fact, 60% to 90% of the energy needed by myocardium originates from the ATP produced by aerobic oxidation of fatty acids. Only 10% to 40% of the energy is generated by glucose glycolysis and lactic acid oxidation. In addition, the production and utilization of ketone body, ornithine, heme, cardiolipin, and ubiquinone are all related to mitochondria [10].

As a vital functional organelle in myocardial cells, the function of mitochondria is key to elucidating the physiological and pathological changes in CVD, and mitochondrial homeostasis is the core element for maintaining myocardial metabolism, function, and structure [11].

## 4. Mitochondrial Homeostasis

Mitochondrial homeostasis is the steady-state balance between mitochondrial biogenesis and degradation. It involves many aspects such as mitochondrial division and fusion [6, 12], mitochondrial crest remodeling [6, 8], mitochondrial biosynthesis [13, 14], mitochondrial autophagy [15, 16], and mitochondrial oxidative stress [9, 17]. Mitochondrial homeostasis refers to the healthy and stable state of mitochondrial content and metabolism for ensuring the stability of cell energy supply and material metabolism. To maintain the integrity of the mitochondrial structure, mitochondrial division and fusion and mitochondrial crest morphology are altered along with changes in intracellular energy supply [12]. Mitochondrial health is maintained through biosynthesis and autophagy degradation to respond to different energy requirements of cells [10, 15]. In addition, ROS in mitochondria can be used as signal molecules to activate redox signal molecules through redox reaction, thus participating in the regulation of intracellular signal transduction [18]. Disruption of mitochondrial homeostasis may cause imbalance of mitochondrial motility, lysis of mitochondrial cristae, disruption of mitochondrial biosynthesis, abnormal degradation of mitochondrial autophagy, and oxidative stress in mitochondria. Therefore, the stable state of mitochondrial structure and function has very important physiological significance for the growth, metabolism, and heredity of organisms [19].

## 5. Mitochondria Dysfunction and Cardiovascular Diseases

Mitochondria are the energy factories of cells, and their main function is to consume oxygen and metabolize three major nutrients (sugars, lipids, and amino acids) to produce CO_2_, water, and energy (ATP) [4]. Cells often need to manage their energy expenditure based on the availability of nutrients and their ability to produce ATP [10]. Disrupted mitochondrial homeostasis will lead to abnormal metabolism of these common substances in the body. Higher organisms need to consume larger amount of energy, and the ATP produced by anaerobic glycolysis is only approximately 1/16 of that produced by aerobic oxidation.

Mitochondria are exposed to various physiological or stress signals, and they produce different signal molecules that affect oxidative stress, apoptosis, autophagy, and inflammation, which are closely related to the occurrence of CVD [11, 16, 20]. The pathophysiological processes of abnormal effects of mitochondria on CVD are reflected in the following aspects: (1) because cardiomyocytes rely on fatty acid-driven oxidative phosphorylation to produce ATP, a decline in the biological efficiency of the mitochondrial network may directly harm the contractility of cardiomyocytes; (2) because Ca^2+^ flow is the core of overall cardiac activity, incapability of the mitochondrial network to regulate Ca^2+^ homeostasis can alter cardiac function; (3) physiological inflammatory homeostasis has a certain protective effect not only on cardiac function but also on vascular filling, but the accumulation of damaged mitochondria in the cytoplasm of cardiomyocytes or endothelial cells can cause pathogenic inflammation; and (4) the integrity of the cardiovascular system is essential for cardiac contractile and circulatory functions. Severe mitochondrial dysfunction and accumulation of damaged mitochondria initiate a series of cell death that eventually leads to pathological damage.

## 6. Mitochondrial Dysfunction and Atherosclerosis

Atherosclerotic (AS) is the main cause of death due to cardiovascular disease. In patients with mitochondrial dysfunction, decreased activity of progressive respiratory chain enzymes, excessive production of ROS, and cumulative mitochondrial DNA (mtDNA) damage or mutations are closely related to the occurrence and development of atherosclerosis [21, 22]. Studies have shown that oxidized low-density lipoprotein (ox-LDL) plays an important role in the occurrence and development of atherosclerosis; ROS produced by mitochondria and its modified ox-LDL are involved in all pathological processes of atherosclerosis [23]. Ox-LDL can slow down the electron transport of mitochondrial respiratory chain by inhibiting the activity of mitochondrial respiratory enzymes and increasing the formation of ROS, thus forming a vicious circle and promoting endothelial injury and atherosclerosis [23]. It was found that when the activity of Mn-SOD (SOD_2_) decreased, mtDNA damage increased in apoE^−/−^ rats, which preceded the formation of atherosclerotic plaques. As oxidative stress in mitochondria increased, atherosclerotic lesions were significantly aggravated [24]. In addition, studies in apoE^−/−^-SOD_2_^+/−^ mice have shown that an increase in mitochondrial ROS not only promoted the formation of atherosclerotic plaques but also increased the susceptibility of the body to atherosclerotic risk factors [25]. Moreover, mtDNA damage caused by DNA repair dysfunction can directly accelerate atherosclerosis in apoE^−/−^ rats and promote diabetic atherosclerotic complications. Furthermore, transient opening of mitochondrial permeability transition pore (mPTP) can depolarize mitochondrial membrane potential, whereas long-term opening of mPTP leads to matrix swelling, rupture of mitochondrial outer membrane, and apoptosis. Both of these changes can promote the occurrence and development of atherosclerosis [21, 22]. In an experiment using wild-type mice, it was found that aging led to increases in IL-6 level and mitochondrial dysfunction. Hyperlipidemia further decreased the mitochondrial function and increased the level of Parkin in the aorta of old mice (16 months of age). Importantly, oral spermidine can enhance the mitotic function of aged hyperlipidemic mice, prevent elevation of aortic IL-6 and Parkin levels, reduce mitochondrial dysfunction, and reduce atherosclerosis formation. Overall, new treatments that improve vascular mitochondrial bioenergetics or reduce inflammation before hyperlipidemia may reduce age-related atherosclerosis [26]. Overall, oxidative stress, inflammatory reaction, and mitochondrial dysfunction play a key role in the formation of atherosclerosis. Mitochondria-targeted antioxidant and anti-inflammatory therapies may have great prospects for the treatment of atherosclerosis [27].

## 7. Mitochondrial Dysfunction and Hypertension

Hypertension is a common CVD in modern society. Many studies have shown that mitochondrial dysfunction is closely related to hypertension [28]. The superoxide anions produced by mitochondria can oxidize the NO released by endothelial cells, decrease the endothelium-dependent vasodilation function, increase vascular force, and increase blood pressure. Uncoupling of mitochondrial oxidative phosphorylation caused by UCP2 gene polymorphism or altered expression is also associated with high blood pressure [29]. In addition, the lack of mitochondrial productivity, calcium overload, and mitochondrial DNA mutations are all involved in the pathological process of arterial hypertension and hypertensive heart disease. Angiotensin II (Ang II) plays an important role in the development of hypertension. Ang II can also inactivate the NO produced by endothelial cell by stimulating the production of mitochondrial ROS, resulting in vascular endothelial dysfunction [30]. Mitochondrial dysfunction is also related to dysfunction of blood pressure regulation center [31]. Related research has confirmed that mitochondrial dysfunction caused by maternally inherited mitochondrial transfer ribonucleic acid (tRNA) mutations is associated with the development of essential hypertension [32]. Otherwise, under the conditions of inflammation, Ang II stimulation, and metabolic syndrome, disturbances in mitochondrial biogenesis and mitochondrial bioenergetics in the brain will lead to the accumulation of ROS, which plays an active role in the pathophysiology of ROS-related neurogenic hypertension [33]. Overall, increased ROS production, decreased ATP production, and calcium overload play an important role in the occurrence and development of hypertension. Moreover, mitochondrial gene polymorphism and mitochondrial tRNA gene mutations are also associated with hypertension.

## 8. Mitochondrial Dysfunction and Myocardial Ischemia-Reperfusion Injury

Myocardial ischemia-reperfusion injury (IR injury) is common in reperfusion therapy after acute myocardial infarction, manifesting as arrhythmia, reduced cardiac systolic function, and other phenomena. Mitochondrial energy metabolism disorder is an important factor causing myocardial IR injury [34]. The main mechanisms include reduced mitochondrial ATP production and excessive ROS production, causing oxidative stress, Ca^2+^ overload, and sustained mPTP opening [18, 35]_._ Excessive ROS production during ischemic myocardial reperfusion is the main cause of myocardial IR injury, and mitochondria are an important source of ROS. On the one hand, increased ROS can damage the mitochondrial membrane system, which affects the mitochondrial membrane potential and disrupts mitochondrial ATP synthesis. On the other hand, mitochondria produce excessive ROS, which causes peroxidation of proteins and lipids and damage to the mitochondrial membrane, further decreasing the activity of the electron transport chain enzymes, which in turn form a vicious circle that eventually leads to cardiomyocyte apoptosis and necrosis [36]. In addition to excessive ROS, myocardial ischemia-reperfusion-induced cell Ca^2+^ overload is an important cause of myocardial IR injury [18, 35]. Persistent opening of mitochondrial mPTP with high permeability also plays an important role in IR injury. This causes the entrance of numerous small molecules to mitochondria, resulting in the swelling of mitochondria, rupture of the outer membrane, collapse of membrane potential, and release of various proapoptotic factors to induce cell apoptosis or death [35]. Taken together, improving mitochondrial function, reducing oxidative stress caused by excessive production of mitochondrial ROS, preventing intracellular calcium overload, and preventing the opening of mitochondrial mPTP are effective measures for the prevention and treatment of IR injury.

## 9. Mitochondrial Dysfunction and Heart Failure

Heart failure (HF) is the final stage of the development of various CVDs, such as myocardial infarction, hypertension, and cardiomyopathy. The relationship between mitochondrial dysfunction and HF is mainly reflected as follows: the disturbance of mitochondrial energy metabolism plays an important role in the occurrence and development of HF. During HF, mitochondrial ATP synthesis decreased, and ROS production increased, whereas the disturbance of energy metabolism in myocardial mitochondria aggravated the disruption of cardiac mechanical function and deterioration of cardiac function. ROS modified myofibrillar protein in the myocardium via oxidation, resulting in a progressive decrease in cardiac contractile function and irreversible cardiac injury [37, 38]. Studies in an experimental HF model have shown that the expression of myocardial mitochondrial biosynthesis factor is downregulated, whereas mtDNA content is reduced, which not only results in reduced mitochondrial biosynthesis but also causes mitochondrial oxidative phosphorylation and reduces the ability of mitochondria to oxidize fatty acids, which leads to deficiencies in myocardial energy production and HF development [39]. In patients with congenital heart disease, damage in mtDNA replication leads to the loss of right ventricular mtDNA, resulting in the progression of heart hypertrophy to HF [40, 41]. Therefore, to prevent mitochondrial damage and maintain the integrity of its function, reducing oxidative stress will be an important strategy in the treatment of HF [41].

In summary, the maintenance of mitochondrial homeostasis is very important in life activities, and mitochondrial dysfunction is closely related to the occurrence and development of CVD. TCM is widely applied in the clinical treatment of CVD, and mitochondria are the intracellular targets of many kinds of drugs. Thus, we propose that TCM can treat CVD by affecting mitochondrial homeostasis.

## 10. Protective Effect of TCM on Myocardial Mitochondria

Mitochondria play a significant role in the regulation of physiological function and pathological process in the cardiovascular system [11]. TCM [42], including the chemical components, single herbs, and compound medicines, can treat CVD by regulating the function of mitochondria, which will be described below.

### 10.1. Chemical Components of TCM

The chemical components of TCM are the substance bases of its pharmacology. The composition of TCM is extremely complex, as each TCM contains many kinds of chemical components. Components that have biological activity and play a role in the prevention and treatment of diseases are known as effective components. Modern studies have shown that the effective components of TCM can protect cardiomyocyte mitochondria in many ways. Several common drugs are summarized in Tables 1–4.

### 10.2. Single Herbs of TCM

Single herbs are a type of TCM. Different single herbs have different curative effects, but they may have the same drug action. A single herb can have many different effects. Previous studies have shown that some single-herb medicines can treat coronary heart disease (CHD) by affecting mitochondrial homeostasis. Several common drugs are summarized in Table 5.

### 10.3. Compound Prescriptions of TCM

Compound prescriptions are the main form of clinical TCM. After determining the treatment based on syndrome differentiation, a compound prescription is formulated by selecting the appropriate drug, determining the dosage, and combining two or more medicines according to the requirements of the basic structure of the prescription. The main objectives of these prescriptions are to enhance drug efficacy, produce synergistic drug effects, control the direction of multifunctional single herbs, expand the scope of treatment, improve drug adaptation to complex conditions, and control the toxic and side effects of drugs.

Clinically, most of the drugs used to treat CHD are compound prescriptions. The regulation of CHD by compound prescriptions involve the whole body, including the heart, brain, liver, kidney, lung, large intestine, muscle, and other viscera, and they improve the structure and quantity of mitochondria in each tissue [39, 74]. In addition, compound prescriptions can treat CHD by protecting mitochondrial function, reducing antioxidant stress, improving mitochondrial lipid metabolism, and exerting anti-inflammatory effect. The compound prescriptions commonly used in TCM are listed in Table 6.

In summary, mitochondria are semiautonomous organelles that integrate the three basic life activities: material metabolism, energy metabolism, and genetic variation; they are also the place for intracellular respiration and energy conversion and participate in various important physiological and biochemical processes. Overall, TCM affects the processes of mitochondrial energy metabolism, apoptosis, and oxidative stress in multilevels via multitargets, and the same category of drugs has certain commonness and individuality. The mechanisms are summarized in Table 7.

## 11. Conclusions and Perspectives

CVD is the leading cause of death in China [2]. CHD is a relatively common type of CVD. At present, CHD has become a major global public health problem; although antithrombosis, anti-ischemia, and lipid-regulating interventional therapies and secondary preventions have been used to improve CHD symptoms and reduce the mortality and HF after percutaneous coronary intervention(PCI), no reflow after revascularization, depression after CHD, CHD complications, and antithrombotic drug resistance still persist as clinical problems that need to be solved. At present, syndrome differentiation via a combination of modern medicine and TCM is the main method for treating CVD in China and abroad [88].

Coronary atherosclerosis or vasospasm leads to decreased myocardial blood perfusion and increased ischemic damage of cells. During the ischemic period, hypoxia causes inhibition of mitochondrial ATP synthesis and oxidative phosphorylation, making it difficult for cells to maintain normal ATP content. At the same time, under the condition of ischemia and hypoxia, excessive metabolites, such as lactic acid, pyruvate, phosphate, and other acids, accumulate in the myocardium and produce symptoms such as angina pectoris or chest tiredness. An evidence showed that mitochondrial dysfunction occurs in the early stage of CHD and mitochondrial autophagy occurs in the late stage, which involves the steady-state dynamic balance of mitochondria [89].

A growing number of studies showed that mitochondria play an important role in the cardiovascular system. Mitochondria can be used as targets for the treatment of CVDs [90]. Mutations in mtDNA affect CVD, leading to hypertension, atherosclerosis, and cardiomyopathy. However, TCM can regulate the structure and function of mitochondria by increasing electron transport and oxidative phosphorylation of mitochondria, thus regulating mitochondria-mediated apoptosis and reducing mitochondrial ROS to treat CVD.

At present, there are multiple forms of TCM used in the treatment of CVD, including its active components, single herbs, and compound prescriptions [3, 42]. One review of 68 randomized controlled trials that included a total of 16171 patients revealed that, compared with blank control or placebo, TCM effectively reduces the severity of angina pectoris and MI; it also lowers blood pressure in patients with hypertension and improves cardiac function in patients with HF [91]. In most studies, the frequency of adverse effects was not higher for TCM than for controls or Western medicine [91]. However, the methodological quality of the majority of included studies was low; further studies using strictly designed randomized controlled trials are necessary to provide strong evidence [92]. Owing to the complexity of CVD pathogenesis, the action mechanism of TCM in the treatment of CVD is difficult to clarify. Moreover, mitochondria are the intracellular targets of many drugs, and there are extensive and complex interactions between mitochondria and related drugs [90].

TCM has the advantages of multitarget and multipathways [42, 93]. A large number of clinical experiences and studies have shown that TCM plays an important role in the prevention, treatment, and prognosis of CVD [93]. Previous studies by our team have shown that circRNAs are closely related to the pathological process of acute coronary syndrome via a mechanism that may be related to the up- or downregulation of circRNAs and miRNAs and the coexpression of circRNA-miRNA [94]. In addition, through literature review, we consider that the circRNA-miRNA-mRNA network may be a new regulatory mechanism for TCM to effectively treat CVD [93]. Besides, TCM has been shown to improve the morphology and structure of mitochondria and participate in a series of metabolic processes, including regulation of energy metabolism, inhibition of apoptosis, and reduction of oxygen free radical production in mitochondria. There is a certain correlation between the intracellular targets of TCM and mitochondria.

Furthermore, we should treat diseases according to syndrome differentiation and reasonably choose TCM based on the basic theory of TCM. Integrating TCM with Western medicine, an unprecedented task in today's world, would provide a new medical model with unique advantages that can play an important role in the treatment of diseases [95]. Some experiments have confirmed the efficacy of combining TCM with Western medicine for the treatment of angina pectoris [96], and evidence for using TCM for the treatment of other CVDs is also increasing [3]. The combination of TCM and Western medicine is an attractive avenue for therapeutic intervention in CVD, and it may be the best scheme for CVD treatment. Thus, developing a strategy for integrating TCM with Western medicine will greatly contribute to human health care [95].

## Figures and Tables

**Table 1:** Regulatory effects of active components of TCM on mitochondria.

Category	Chemical components of TCM	Monomer source	Molecular formula	Mechanism of action	Reference
Restoratives for invigorating qi	Ginsenoside compound K	*Radix ginseng*	C_36_H_62_O_8_	Inhibition of nuclear factor-B*κ*, p38, and JNK MAPK pathways	Lu et al. [43]
	Astragaloside IV	*Radix Astragali*	C_41_H_68_O_14_	Regulation of NF-*κ*B/PGC-1*α* signaling-mediated energy biosynthesis	Zhang et al. [44]
				Downregulation of miR-23a and miR-92a-activated PI3K/AKT and MAPK/ERK signaling pathways	Gong et al. [45]
				Stimulation of fatty acid *ß*-oxidation and improvement of mitochondrial function	Dong et al. [46]
	Astragalus polysaccharides	*Radix Astragali*	C_10_H_7_C_l_N_2_O_2_S	Inhibition of apoptosis	Liu et al. [47]
	Salidroside	*Rhodiola crenulata*	C_14_H_20_O_7_	Activation of a mitochondria-associated AMPK/PI3K/Akt/GSK3*β* pathway	Zheng et al. [48]

Restoratives for nourishing yin	Ophiopogonin D	*Radix Ophiopogonis*	C_44_H_70_O_16_	Antioxidant and antiapoptotic effects	Huang et al. [49]
	Ecliptal	*Eclipta alba*	—	Activation of the Wnt-pathway and alteration of AKT signaling	Yang et al. [50]

Restoratives for nourishing yang	Velvet antler	*Cornu cervi pantotrichum*	—	Regulation of the PI3K/Akt signaling pathway and mitochondrial membrane potential	Xiao et al. [51]
	Icariin	*Herba Epimedii*	C_33_H_40_O_15_	Activation of sirtuin-1/FOXO1 signaling and improvement of mitochondrial membrane homeostasis	Wu et al. [52]

**Table 2:** Regulatory effects of the active components of traditional Chinese medicine on mitochondria for promoting blood circulation and removing blood stasis.

Chemical components of TCM	Monomer source	Molecular formula	Mechanism of action	Reference
Panax notoginseng saponins	*Radix Notoginseng*	C_47_H_80_O_17_	Attenuation of oxidative stress and cardiomyocyte apoptosis	Zhang et al. [53]
				Zhou et al. [54]
Notoginsenoside R1	*Radix Notoginseng*	C_54_H_92_O_24_	Elevation of mitochondrial ATP synthase *d*-subunits	He et al. [55]
Salvianolic acid A	*Radix Salviae Miltiorrhizae*	C_26_H_22_O_10_	Promotion of myocardial mitochondria biogenesis	Zhang et al. [56]
Salvianolic acid B	*Radix Salviae Miltiorrhizae*	—	Improvement of mitochondrial biogenesis	Pan et al. [57]
Tanshinone IIA	*Radix Salviae Miltiorrhizae*	C_19_H_18_O_3_	Upregulation of 14-3-3*η*, prevention of mPTP opening, and inhibition of apoptosis	Zhang et al. [58]
Dihydronortanshinone	*Radix Salviae Miltiorrhizae*	—	Anti-inflammatory effect via the NF-*κ*B, mitochondrial ROS, and MAPK pathways	Wu et al. [59]
Curcumin	*Rhizoma Curcumae Longae*	C_21_H_20_O_6_	Antioxidant and anti-inflammatory activities	Li et al. [60]
			Mitochondrial stress and substrate switching inhibition	

**Table 3:** Regulatory effects of the active components of interior warming medicines on mitochondria.

Chemical components of TCM	Monomer source	Molecular formula	Mechanism of action	Reference
Flavonoid glycosides	*Fenugreek*	—	Regulation of glycolipid metabolism	Luan et al. [61]
	*Rhizoma Zingiber*	C_17_H_26_O_4_	Improvement of ectopic lipid accumulation, mitochondrial dysfunction, and insulin resistance	Liu et al. [62]

**Table 4:** Regulatory effects of the active components of other traditional Chinese medicines on mitochondria.

Type of TCM	Monomer source	Molecular formula	Mechanism of action	Reference
Triptolide	*Tripterygium wilfordii*	C_21_H_28_O_6_	Regulation of mitochondrial membrane permeabilization	Xi et al. [63]
Oxymatrine	*Radix Sophorae Flavescentis*	C_16_H_26_N_2_O_2_	Inhibition of cardiac apoptosis and oxidative stress	Zhang et al. [64]
Epigallocatechin gallate	*Green tea*	C_22_H_18_O_11_	Inhibition of deterioration of mitochondrial structure and function by OMA1	Nan et al. [65]
Cyclovirobuxine D	*Buxus microphylla Sieb*	C_26_H_46_N_2_O	Antioxidant effect and promotion of mitochondrial biogenesis	Guo et al. [66]
Tetrandrine	*Radix Stephaniae Tetrandrae*	C_38_H_42_N_2_O_6_	Regulation of glycolysis and energy metabolism	Zhang et al. [67]

**Table 5:** Regulatory effects of single-herb traditional Chinese medicines on mitochondria.

Type of TCM	Single herb	Mechanism of action	Reference
Restoratives for invigorating qi	*Radix Astragali*	Promotion of mitochondrial bioenergetics	Huang et al. [68]
Restoratives for invigorating qi	*Rhodiola rosea*	Promotion of mitochondrial biogenesis and functions	Zhuang et al. [69]
		Antioxidant and anti-inflammatory activities	Zhou et al. [70]
Heat clearing Chinese medicinal herbs	*Silybum marianum*	Mitigation of oxidative stress and attenuation of reactive fibrosis via TGF*ß*1/T*ß*Rs/SMAD2/3 signaling	Vilahur et al. [71]
Invigorating the blood and removing blood stasis	*Salviae Miltiorrhizae Radix et Rhizoma*	Activation of the Nrf2-mediated antioxidant defense system	Li et al. [72]
The interior warming Chinese medicinal herbs	*Cortex Cinnamomi*	Upregulation of mitochondrial biogenesis	Song et al. [73]

**Table 6:** Regulatory effects of compound prescriptions on mitochondria.

Name of compound prescription	Components of compound prescription	Mechanism of action	Reference
Shengmai formula (SM)	*Radix ginseng* and *Radix Ophiopogonis*	Protection of cardiomyocytes against hypoxia	Wang et al. [75], Yu et al. [76]
		Induction of mitophagy and modulation of mitochondrial dynamics	
Shenxian-Shengmai oral (SXSM)	*Red Radix ginseng, Herba Epimedii, Fructus Psoraleae (salted), Fructus Lycii, Herba Ephedrae,* etc.	Antioxidant effect, promotion of SOD activity, elevation of GSH content, and reduction of intracellular ROS levels	Zhao et al. [77]
YiXin-Shu (YXS)	*Ginseng, Radix Astragali, Salvia miltiorrhiza, Ophiopogon, Ligusticum,* etc.	Upregulation of endogenous nuclear receptors (LXR*α*, PPAR*α*, PPAR*β*, and ER*α*) as well as suppression of apoptosis and oxidative stress	Zhao et al. [78]
Shengmai San (SMS)	*Panax ginseng, Ophiopogon japonicus,* and *Schisandra chinensis*	Improvement of mitochondrial lipid metabolism, restoration of mitochondrial structure and function, and promotion of mitochondrial biogenesis via the Sirt1/PGC-1*α* pathway	Tian et al. [79], Lu et al. [80], and Li et al. [81]
QiShenYiQi Pills (QSYQ)	*Radix Astragali, Salvia miltiorrhiza, Panax notoginseng,* etc.	Regulation of energy metabolism and elevation of mitochondrial content and biogenesis via PGC-1*α* activation	Lin et al. [82], Yu et al. [83], and Lin et al. [84]
Shexiang Baoxin Pill (SBP)	*Moschus, Radix Ginseng, Calculus Bovis, Styrax, Cortex Cinnamomi, Venenum Bufonis,* and *Borneolum Syntheticum*	Anti-inflammatory and antioxidant effects, improvement of lipid metabolism, protection of mitochondrial function, and upregulation of AMPK and PGC-1a expression	Wei et al. [85]
Qiang-Xin 1 formula	*Astragalus, Poria, Schisandra, Salvia miltiorrhiza,* etc.	Prevention of sepsis-induced apoptosis	Xu et al. [86]
Tongxinluo capsule (TXL)	*Radix ginseng, Hirudo, Scorpio, Radix Paeoniae Rubra,* etc.	Anti-inflammatory effect and improvement of lipid metabolism	Zhang et al. [74] Ma et al. [87]

**Table 7:** Action mechanisms of TCM on mitochondria.

Action mechanism	Chemical components of TCM	Single herb	Compound prescription
Mitochondrial structure	Velvet antler, icariin, tanshinone IIA, triptolide, and epigallocatechin gallate	—	Shengmai San
Mitochondria biosynthesis	Ecliptal, salvianolic acid A, salvianolic acid B, and cyclovirobuxine D	*Radix Astragali, Rhodiola rosea, Cortex, Cinnamomi*	QiShenYiQi pills
Mitochondrial function	Flavonoid glycosides, 6-gingerol, and epigallocatechin gallate	*Rhodiola rosea*	Shengmai formula, Shengmai San, and Shexiang Baoxin pill
Anti-inflammatory effect	Ginsenoside compound K, astragaloside IV, dihydronortanshinone, and curcumin	*Rhodiola rosea*	Tongxinluo capsule (TXL), Shexiang Baoxin pill
Inhibit apoptosis	Astragalus polysaccharides, ophiopogonin D, and oxymatrine	—	YiXin-Shu, Qiang-Xin 1 formula
Antioxidation	Ophiopogonin D, panax notoginseng saponins, oxymatrine, cyclovirobuxine D, and curcumin	*Rhodiola rosea, Silybum marianum, Salviae, Miltiorrhizae Radix et Rhizoma*	Shenxian-Shengmai oral, YiXin-Shu, and Shexiang Baoxin pill
Energy metabolism	Salidroside, notoginsenoside R1, and tetrandrine	*Radix Astragali*	—

## Data Availability

This review article is based on some previous studies of the authors on the relationship between mitochondria and cardiovascular diseases and the role of traditional Chinese medicine in the treatment of cardiovascular diseases. Previously reported studies were used to support this study and are available at doi: 10.1155/2015/143145, doi: 10.1155/2020/1584052, doi: 10.1155/2020/8048691, etc. In addition, the results obtained through extensive literature review provide strong support for the article. These prior studies are cited at relevant places within the text as references.
